# Use of delivery drones for humanitarian operations: analysis of adoption barriers among logistics service providers from the technology acceptance model perspective

**DOI:** 10.1007/s10479-023-05307-4

**Published:** 2023-04-19

**Authors:** David Edwards, Nachiappan Subramanian, Atanu Chaudhuri, Piera Morlacchi, Wen Zeng

**Affiliations:** 1 University of Sussex, Brighton, United Kingdom; 2 Nottingham University Business School China, University of Nottingham Ningbo, Ningbo, China; 3 University of Sussex Business School, University of Sussex, Brighton, United Kingdom; 4 Durham University Business School, Durham University, Durham, United Kingdom; 5 Zheshang Development Group Co. Ltd., Hangzhou, China

**Keywords:** Humanitarian logistics, Drones, Adoption barriers, China

## Abstract

Delivery drones are yet to be adopted as a systematic delivery system for humanitarian operations but have the potential to substantially increase the efficiency and effectiveness of future delivery options. Thus, we analyse the impact of factors affecting the adoption of delivery drones by logistics service providers for humanitarian operations. A conceptual model of potential barriers to adoption and development is created using the Technology Acceptance Model theory involving security, perceived usefulness, perceived ease of use and attitude as factors that affect the intention to use. We validate the model using empirical data collected from 103 respondents by the 10 leading logistics firms located in China between May and August 2016. through a survey to examine factors currently affecting the intention/non-intention to adopt delivery drones. The results show that ease of use and addressing key security considerations about the drone, the delivery package and the recipient are crucial for adopting the technology as a specialized delivery option for logistics service providers. This is the first study of its kind and contributes to understanding the operational, supply chain and behavioural factors in the adoption of drones by logistics services providers for humanitarian operations.

## Introduction

Drones having autonomous flight capabilities is an advanced version of unmanned aerial vehicles (UAVs) could become an important transportation system to fulfil logistics needs during emergency situations and humanitarian operations where conventional logistical methods are not effective. Humanitarian operations refer to efforts to provide aid and assistance to affected populations for both natural and man-made disasters, which can be both gradual or occur from a sudden onset. Slow onset natural disasters include famine and drought, while ‘sudden onset’ disasters include tsunami, or forest fires. Slow onset man-made disasters include political or refugee crises while sudden-onset include terrorist attacks (Rao et al., 2016; Starr & Van Wassenhove, 2014; Altay and Narayanan, 2022).

UAVs are aircraft, or more generally robotic systems, that can be piloted remotely or flown autonomously. They have many applications. For example, UAVs could be useful for disaster relief, infrastructure improvements, transportation and delivery of medical supplies (e.g., medicines, blood, and lab reagents), traffic monitoring, wildlife management, and criminal investigations (Erdelj et al., 2017). In the last five years, the use of UAVs for medical supplies and aid in health supply chains has become more common (Maghazei et al., 2022). However, drones have been recognized as one of the most promising technologies to improve disaster response and relief operations (Maric et al., 2021). When a disaster occurs, drones can be used to provide better situational awareness, locate survivors, perform structural analysis of damaged infrastructure, deliver needed supplies and equipment, evacuate casualties, and help extinguish fires – among many other potential applications (American Red Cross 2015; Maric et al., 2021). Indeed, drone enabled systems could significantly improve agility in humanitarian logistics since they do not need any pre-existing path to fly. Therefore, if roads are blocked due to a natural disaster, drones can easily be used to serve the disaster affected region (Chowdhury et al., 2017). Although drones can potentially be used to deliver lightweight goods, such as vaccines, during humanitarian disasters, use of drones by logistics service providers for such operations will depend on further detailed analysis, and consideration of factors such as the fragility of the goods and disruption of the local conditions caused by the disasters (Comes et al., 2018; Rejeb et al., 2021).

The use of drones for both commercial and humanitarian purposes has so far received limited attention by the logistics service providers, possibly due to technological constraints in terms of the weight that can be carried, limited battery life, and interference with commercial jet operations (Rao et al., 2016). In the recent review it is emphasised to explore the capabilities of drones and the impact of their technical features on the performance of humanitarian operations (Rejeb et al., 2021). Moreover, concerns about how drones relate to universal humanitarian principles, such as impartiality, dignity, the prioritization of safety above all else, sensitivity to conflict, and to collecting, using and storing data responsibly, have been raised in recent years (Meier, 2014; USAID 2017; Van Wynsberghe et al. 2018). There is growing concern that any logistics service provider with a drone facility could call themselves a disaster relief operator. This has fuelled calls for ways to ensure that operators reach a required professional standard (Maric et al., 2021). In 2014, the Humanitarian UAViators Network, a platform to discuss best practices in the field, released a code of conduct addressing regulators and communities’ concerns. It is hoped that the code will be incorporated into national regulations, but as yet the legal position of drones for commercial and humanitarian purposes is still unclear (Rao et al., 2016; Van Wynsberghe et al., 2018). Specifically during pandemic there were investigative studies in collaboration with Red Cross organisation and utility drone manufacturer to understand how drones can be used to distribute the viral testing kits to the infected people (Kunovjanek & Wankmuller, 2021). Moreover, many proof of concepts to overcome infrastructural and organisational barriers post pandemic to handle medical items delivery with the use of drones have been explained by Sarker et al. (2021). In addition, a few case studies call for promoting innovation and use of technology to make the humanitarian supply chain flexible (Besiou & Van Wassenhove, 2020; Evanthia, 2019).

One way to professionalize drone operations is its adoption by appropriate third party logistics service providers., it is not apparent whether logistics service providers will adopt drones and find those suitable for deploying during humanitarian operations. In particular, the study by Maghazei et al. (2022) emphasised the need for fit between technology and other factors such as economic and strategic factors, operational and supply chain factors and organisational and behavioural factors. In addition the study by Maghazei et al. (2022) articulated the huge gap in operations management literature in understanding the operational, supply chain and behavioural factors with respect to drone technology. Therefore, this study is essential to understand the readiness of logistics service providers to adopt drones for humanitarian operations and to explain the role of innovative technologies used in humanitarian operations (Gunasekaran et al., 2018; Heaslip, 2018).

Therefore, this study aims to answer the related research questions:

1. What are the key factors (operational, supply chain and behavioural factors) that affect drone enabled delivery systems in humanitarian operations; and.
2. To what extent do the above factors impact third-party logistics service provider’s intention to adopt drones in humanitarian operations?

We develop and use a research model based on theories and applications of the Technology Acceptance Model (TAM) and its developments and extensions (Davis, 1985, 1989; Davis et al., 1989; Venkatesh & Bala, 2008; Marangunic and Granic 2015) to explore barriers in the adoption of delivery drones by Chinese third party logistics providers (3PLs). We use structural equation modelling to assess the impact of the different factors on the intention to use drone-enabled delivery systems. With China shifting its position from trying to catch up with the rest of the world to becoming a global frontrunner in the drone industry, and a pacesetter in its evolution in terms of standards and regulation, our study of 10 major third party service logistics companies provides an important setting to explore the factors that influence the adoption of drone delivery systems for humanitarian purposes.

The next section of the paper presents a literature review including humanitarian operations and drone use in delivery logistics. The research model that was developed and applied in this study is then explained. Section 4 describes the methodology, data analysis and findings, followed by a discussion of the analysis, implications and limitations.

## Literature review

### Humanitarian operations and drones

Humanitarian operations is defined as the processes and systems involved in mobilizing people, resources, skills and knowledge to help vulnerable people affected by disasters – both natural and man-made, slow and sudden onset (Van Wassenhove, 2006; Besiou & Van Wassenhove, 2020). Early studies on humanitarian operations were more concerned about three aspects (preparedness, response and collaboration (Tomasini & Wassenhove, 2009), and the possibility of involving supply chain principles (Oloruntoba & Gray, 2006). The studies found that agile supply chain principles are suitable to address humanitarian operations needs, such as lean principles and performance measures for upstream donors and agile information systems aid for recipients on the downstream side (Oloruntoba & Gray, 2006). In particular the coordination and use of technology of resource dispatching and rescue operations within downstream activities are understudied (Farahani et al., 2020).

Even though the field received serious attention from supply chain researchers over a decade, many open challenges still persist in the humanitarian operations context, specifically in the last mile of disaster relief operations (Rabta et al., 2018). The study by Rabta et al. (2018) identifies the need of delivery through drones when accessibility using trucks and helicopters is not possible. In addition, the study proposes an optimization model for drone delivery of light-weight items, such as vaccines and water purification tablets, to disaster prone areas with the objective of minimizing the total travel distance; however, this does have constraints such as payload and energy. The study also evaluated various priority policies that can reduce time and cost in different scenarios.

Similarly, Chowdhury et al. (2017) developed an integrated facility location-inventory allocation model for a disaster affected region where drones can be considered as a potential mode of transportation to transport emergency supplies to the demand points. Mosterman et al. (2014) explored possible systems that may aid in disaster relief scenarios involving the coordination of a range of actors; these include human, UAV, and wireless network automation (WSN). Such systems could carry out important tasks in the domains of situational awareness and search and rescue. A recent review article by Erdelj et al. (2017) emphasizes the use of wireless sensor networks and multi-UAVs for natural disaster management. In particular the focus of the review is to improve the wellbeing of people and move towards the proposal of a complete disaster management system.

Drones have numerous applications in the humanitarian context (USAID 2017; Rejeb et al., 2021). They are tested as a tool in health supply chains, particularly in situations where time is critical in delivery, such as for emergency responses to viruses, search and rescue after a landslide, tsunami or earthquake, and quick surveying and mapping for flood mitigation and camp management. Drones may also offer a cheaper alternative to conventional delivery systems, such as aircraft and trucks, for the shipment of high-frequency, low-weight items such as cold chain products (e.g., vaccines) and other medical supplies. Tatham et al. (2017) provided an overview of how long endurance remotely piloted aircraft systems can provide support in humanitarian operations. Specifically, they can fly over the affected areas and swiftly provide the resultant photographic information to the National Disaster Management Organization. The authors also explained how such an aircraft could have provided a better response to a disaster like Cyclone Pam, which struck Vanuatu. In the recent review, Besiou and Van Wassenhove (2020) envisaged the need for drones in future to make humanitarian operations more efficient and effective based on the successful pilot studies to carry blood to rural areas during humanitarian crisis. Typically technological innovations in future would reduce the suffering of beneficiaries at the lowest cost to overcome the funding constraints prevailing in humanitarian aid operations.

### Drone use in delivery logistics

Some logistics companies have already tried to use drones to deliver parcels and other light-weight items. However, assessment of the potential of drone-enabled delivery systems is under-researched, especially so in rapidly developing economies such as China (Chase et al., 2015). Although human labour is the common method of delivery and proves cost effective in developing economies, it is not always effective in the delivery process, especially in densely populated urban and remote rural environments. Volume and customer service now make it necessary for logistics companies to create new delivery systems. Recently, drone-empowered systems have become widely used tools in many areas, such as in taking aerial photographs, news reporting and environmental monitoring for example (Bowden, 2013; Chase et al., 2015).

Traditional pickup and delivery systems in regular supply chains present two different classes of problems. The Swapping Problem, involving categorization issues and wrong package pick up, characterizes the first many-to-many problem. The second, a one-to-one problem, where drone systems can have an impact, is in replacing courier operations or door-to-door transportation services (Berbeglia et al., 2010). Delivery by labour force can be prone to late delivery and environmental pollution. In contrast, delivery by drones may offer some affordances. Firstly, the use of drone is less limited by space, and it can suit many types of terrain, especially in some remote rural areas (Perez et al. 2017; Evanthia et al. 2019). Secondly, because they can fly from origin directly to the destination, and are not affected by the traffic jams, drone systems have advantages in delivery speed, and lastly, energy consumption is far less.

Although there are potential limitations, including limited ability to carry only light-weight cargo, constraint of battery life, limited ability to operate in adverse weather conditions (such as strong winds, rain, and storms) and interference with commercial jet operations, drone systems have been tried by different companies worldwide. For example, Amazon, DHL and Google are pioneering drones as a new type of delivery mechanism for smaller parcels (Edwards & Subramanian, 2014; Rao et al., 2016). The potential to deliver medicines to remote rural areas has also been explored by companies and humanitarian organizations worldwide during COVID 19 pandemic, with new start-ups and established players involved in the development of new UAV-enabled systems (Sarker et al., 2021; Kunovjanek & Wankmuller, 2021). Moreover a recent review of humanitarian drone suggest to investigate the best operational, supply chain and organisational factors for effective and ethical utilisation of drones in disaster management (Rejeb et al., 2021).

## Conceptual model

We develop and use a research model to explore the barriers to the adoption of delivery drones by Chinese 3PLs. We draw from theories and models of technology acceptance and use (Davis, 1989; Venkatesh et al., 2003), and their applications, to study a variety of technologies across different settings (Venkatesh et al., 2016) to develop the conceptual model. From the organisational perspective, a recent review suggested to apply prominent technology adoption model to understand the usage challenges in humanitarian operational contexts (Rejeb et al., 2021). Based on the purpose of this study and the review of relevant articles in the literature, we discuss the factors that may affect the adoption of drone delivery systems by logistics service providers. A summary research model and hypotheses are developed to understand how users come to accept and their intention to use drone technology.

### Technology acceptance models

Theory and models of technology acceptance and use are helpful to understand technology integration into users’ private and professional life. The Technology Acceptance Model (TAM), initially developed by Davis (1985, 1989) based on the Theory of Reasoned Action (TRA) and Theory of Planned Behavior (TPB), focuses on predicting users’ attitude towards using a new technology. Davis et al. (1989) later developed TAM by adding two critical variables, perceived usefulness and ease of use respectively, as key factors that determine whether users accept the new technology or not. Further development and extension of TAM were proposed and tested. For instance, in addition to the connection between perceived usefulness, perceived ease of use, attitude towards use, and behavior intentions and technology adoption, relevant external variables are considered in key extensions of TAM. Moreover, Venkatesh et al. (2003) proposed an integrated model called the Unified Theory of Acceptance and Use of Technology (UTAUT), and developed it further in collaboration with other scholars (Venkatesh et al., 2016).

In the development of our research model, we considered and reviewed different versions and extensions of TAM and their applications across different technologies, settings, groups of users, and tasks (Marangunic and Granic 2015; Venkatesh et al. 2016). In particular as per recent technological adoption reviews the users attitude is often considered as the predominant factor in explaining the technology adoption process which is a function of perceived ease of use and usefulness (Behl et al., 2022). TAM model is selected in this study where TAM models have been used to explain the acceptance and adoption of various types of new technologies, such as pre-prototypes of information systems (Venkatesh and Davis 2004), processing software (Shapka & Ferrari, 2003), online shopping (Vijayasarathy, 2004; Sivo et al., 2007), biometrics (Miltgen et al., 2013), RFID-enabled services (Pramatari & Theotokis, 2009), and robotic-assisted surgery (BenMessaoud et al., 2011). Moreover, we evaluated alternative models to analyse the factors shaping user intentions at different stages of technology adoption and use such as the ‘Continuous Usage Intention’ (Bhattacherjee & Lin, 2015). For instance, following Bhattacherjee and Lin (2015), as technology acceptance and continuous usage are conceptually and temporally distinct behaviours, we assume that at an early stage of experience with drone technology (e.g., when individuals are completely unfamiliar with the technology), TAM is an adequate model to estimate attitude towards use and adoption. For example, recent applications of TAM include mobile-based agricultural extension service (Verma et al. 2018), sport brand apps (Byun et al., 2018), mobile learning (Al-Emran et al., 2018), and augmented reality at the point of sale (Rese et al., 2014); these demonstrate the validity of TAM, even for modern technologies. In addition, the use of well accepted theories and models haven’t challenged TAM for studying adoption of technologies by individual users in the organisation. Moreover, from the psychological stand point of view users tend to behave differently when measured in terms of rewards while using technology and this has been validated in the previous studies (Behl et al., 2022).

### Research model

This paper draws on different technology acceptance models and existing studies to develop a research model that is appropriate to explore the adoption barriers of drone-enabled delivery systems in Chinese logistics companies. For instance, UTAUT has a strong focus on Performance Expectancy (PE) that relates to expected gains in an individual’s job performance. Because we are exploring perceived barriers to adopting an untested technology in relation to established delivery systems rather than individual expectations of impacts on job performance, we evaluated and concluded that Perceived Usefulness (PU) and Perceived Ease of Use (PEOU) are more relevant than PE. In particular ,rather than focusing on expectations of possible affordances for individuals’ performance by using UTAUT, we focused on ease of use issues, such as safety, that are crucial to evaluating the barriers to using drones. Therefore, security is considered from the customer viewpoint, i.e., using drones for delivery is safe, including personal safety, parcel safety and privacy security. The research variables that constituted the research model are as follows.

*Efficient operations:* Logistics companies will need to consider the efficiency of using new technology. This factor is not only related to customer’s satisfaction but also greatly influenced by costs. Delivery systems need to outperform existing systems in terms of customer service. Therefore, it is necessary to consider both delivery efficiency and cost efficiency.

*Security:* The literature suggests that security is a major concern and barrier to the usefulness and efficiency of delivery drones. Data privacy is a customer concern, such as ensuring that information that will be captured during the delivery is secure. Therefore, in order to satisfy their customers, companies should seriously consider how to protect the customer’s personal information and the parcels’ safety. Drone systems are prone to attack and insecure delivery since they cannot identify the receiver of a package (Insinna, 2014). Moreover, the durability of drones is a major concern (Paul, 2015), parcels need to be protected and secure delivery situations created so that customers have confidence in the drone delivery service. Therefore, we explore whether using drones for delivery is safe and secure, including personal safety, parcel safety and privacy security. Insecure delivery situations will seriously diminish the perceived usefulness and efficiency of delivery drones.

*Perceived usefulness:* Perceived usefulness here can be understood as the level of people’s belief that the new technology would improve performance (Davis, 1989; Davis et al., 1989) perceives usefulness as a mutual improvement that could benefit both employee job performance and institutional performance. In our case, we seek to measure whether logistics companies believe delivery drones will bring efficiency, convenience and safety to users, and whether they think it is useful for both companies and beneficiaries in the humanitarian operations. In general, previous studies have proved that higher levels of perceived usefulness will lead to significant improvement to the individual and the beneficiary (Darrow, 2015).

*Perceived ease of use:* Perceived ease of use can be defined as the level of people’s belief that the new technology would not need a lot of effort and will be easier to use (Davis, 1989). In particular Davis et al. (1989) states the construct “ease of use” as freedom from difficulty or great effort. If a new technology is perceived to be effortless to use compared to others, most people will choose it. Directing an autonomous drone to a particular location with the specific object to be delivered is perceived to be easy compared to planning delivery of goods using other methods, particularly during humanitarian operations, logistics service providers are expected to use it.

*Attitude towards use:* Attitude can be understood as a person’s ideas towards a new concept or innovation. It includes subjective evaluation and will result in behavior tendencies (Doob, 1947). As discussed by Dwivedi et al. (2019), attitude corresponds to an employee’s positive or negative feeling when performing the target behaviour. Cognizance and affection are two factors directly determining attitude (Fishbein, 1976; Derwik & Hellström, 2017), and both are deemed as key components in the Theory of Reasoned Action. Here, attitude towards use is defined as the extent to which users prefer to use drones for delivery and regard it as a useful method. A recent review on acceptance theories reveals the direct positive impact on attitude towards use on behavioral intention to use (Dwivedi et al., 2019).

*Behavioural intention to use:* After developing attitude towards use, people may already have a clear realization of their goals. They have to decide whether to accept or reject new concepts. If they accept, actual behaviour will be the next step. On the other hand, they will not put the concept into action if they reject it. Therefore, behavioural intention to use is defined as users’ intent to use drones for delivery during humanitarian operations. If a logistics planner’s responsibility is to ensure that the right goods are delivered in the right quantities to the affected areas during humanitarian operations, and it is not possible to use other forms of transport effectively to reach the locations, the planner is likely to consider the use of drones as an option. If the planner has developed a positive attitude towards use, he or she is likely to develop a positive behavioural intention to deploy drones.

We develop a series of hypotheses based on the research variables established in the literature review. First, whether the new technologies for efficient operations will have an impact on the perceived usefulness. We have noted the operational cost implications of drone delivery systems, such as equipment costs, labour and expertise; legal costs are seen as potential efficiencies. Financial limitations of various kinds create risk and therefore negatively impact on the perception of ease of use of delivery drone systems. Logistics companies should consider both delivery efficiency and cost efficiency in order to satisfy users. In addition there is a strong evidence in the literature suggesting the emerging technologies such as drones and algorithmic models would standardise the process of handling post disaster operations (Behl and Dutta, 2019). According to Darrow et al. (2015), one type of drone designed by DJI, the Chinese drone manufacturer that controls 70% of the worldwide market and chosen by Walmart, can only fly for 18 min, and the maximum payload is around 6.8 kg. In order to deliver over long distances, logistics companies will need to consider creating distributed locations to be used for battery charging as well as goods storage (Darrow, 2015). Due to this short battery life, logistics companies have to tolerate costs including storage warehouses, standby drones and so on. In addition, Rejeb et al. (2021) suggest to assess the cost savings achieved by integration of drones in humanitarian operations. Also in terms of perceived usefulness it is vital to understand the reduction of cost for potential humanitarian relief efforts with the use of drones. Therefore, the relationship between efficiency and perceived usefulness is as follows:

#### H1

The efficient operations of delivery drones will significantly influence the perceived usefulness of the new technology.

We have described the security and safety problems involved in drone delivery in terms of privacy, recipient authentication and durability as significant issues. The main concerns come from the problems of privacy and actual delivery time (Lotz, 2015). In the process of delivery, drones have to carry cameras that are used for flying the system and send information back to operators for recording. The resulting data will pose a privacy threat for clients and bystanders. As with all data privacy issues, companies will need to consider the full range of customer assurances and counter-measures. Also broadly under safety remit there is a significant difference in the development of drone law and privacy among developed and emerging economies (Seharwat, 2020). Moreover, Komasova (2021) reported the ignorance of theorization of privacy regarding drone use and privacy conceptualisation from the public perception point of view. We hypothesize that these issues will influence perceived usefulness of drone delivery systems. The hypothesis is:

#### H2

The security of using delivery drones will significantly influence the perceived usefulness of the new technology.

In order to create a safe and secure service, companies will have to be expert in the use of drones. According to Daud (2015), researchers have designed a type of drone that uses software to detect path barriers; these drones can fly at a speed of 30mph whilst being able to avoid obstacles detected at least ten metres away. The core part is algorithm driven rather map driven. The situation is by no means perfect and systems have to be further developed to allow drones to fly safely at speed. There is also uncertainty concerning privacy laws and the availability of viable insurance cover for invasion of privacy, personal injury or property damage. Insurance companies are still in the process of developing insurance plans for drones. Standards and regulations related to drones are key issues in their development and use in different countries. This consideration establishes the following hypothesis:

#### H3

The security of using delivery drones will significantly influence the perceived ease of use of the new technology.

Perceived usefulness is related to how helpful a technology is Davis et al. (1989). Specifically there are several potential humanitarian and medical uses of drones such as search and rescue operations, small scale emergency mapping, locating survivors in the rubbles, logistics and emergency delivery of medical supplies and equipment (Evanthia et al., 2019). However in terms of ease of use a few concerns have been reported such as humanitarian drones over conflict zones, legal issues and privacy over data collection and data protection. Hence we postulate, perceived ease of use will directly affect people’s sense of how useful a technology is. Therefore, the hypothesis is built as follows:

#### H4

The perceived ease of use of the delivery drones will significantly influence the perceived usefulness of the new technology.

Research (see: Agarwal and Prasad 1999) supports the relationship between perceived usefulness and positive attitude towards use. In the case of humanitarian operations the use of drones will ensure transparency and accountability for the noble causes championed by the third party logistics service providers. Hence innovation and optimism with respect to perceived usefulness of delivery drones will significantly influence the attitude of the people in the third party logistics service providers (Evanthia et al., 2019). Hence, we establish the hypothesis:

#### H5

The perceived usefulness of the delivery drones will significantly influence the attitude towards new technology use.

According to Davis et al. (1989), perceived ease of use refers to the effort needed to use a technology. If people use a new technology and find that it requires less time to get the same result compared with a previous approach, we can expect that they will choose to use that new technology. In the case of drones there no concerns raised related to ease of use other than the technical limitations it has right now such as its performance in harsh climate, battery longevity, data transfer mechanism, payload capacity and flight endurance (Rejeb, 2021). Therefore, we postulate the following:

#### H6

The perceived ease of use of the delivery drones will significantly influence attitude towards new technology use.

Based on previous technology, acceptance studies perceived that usefulness not only has a direct influence on behavioural intention but is also indirectly moderated through personal attitudes. The direct link between perceived usefulness and intention to use is based on the performance of a new technology. In particular technology such as drone are getting smarter and it will substantially influence the logistics service providers behaviour intention towards use on large scale humanitarian rescue and recovery operations in the future. Already several other emerging technologies such as artificial intelligence’s perceived usefulness had a significant impact on the behavioural intention of the user and beneficiaries (Behl et al., 2022). Therefore, we hypothesize the following:

#### H7

The perceived usefulness of the delivery drones will significantly influence the behavioural intention of new technology use.

Attitude towards use is located between people’s beliefs and the intention. Humanitarian operation managers are more concerned with the loss of human life hence the attitude toward delivery drone use will significantly influence their behavioural intention of the new technology use (Rejeb, 2021). A positive attitude is reflected in positive behavioural intention.

#### H8

Attitudes towards delivery drone use will significantly influence the behavioural intention of new technology use.

**Fig. 1:**
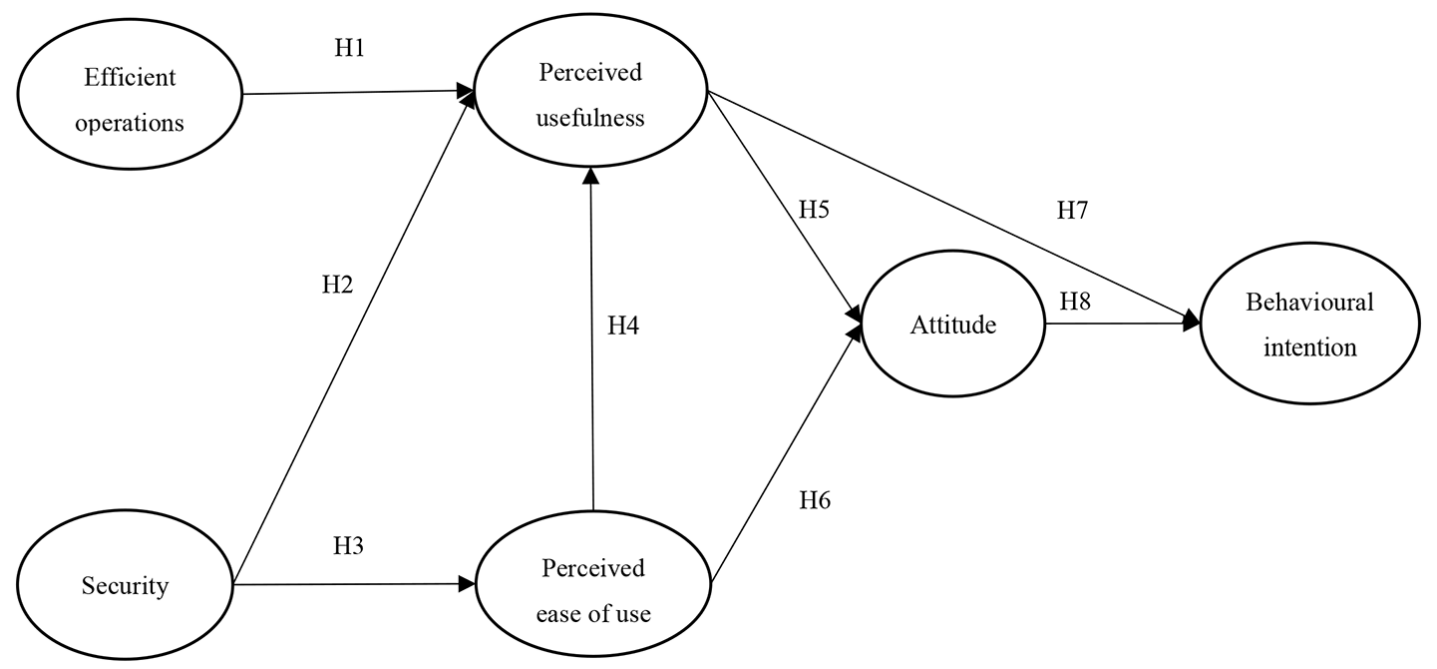
Research Model

## Method

The study was conducted in 2016 using a quantitative research approach and data collected through questionnaires. As per the study by Maghazei et al. (2022) Chinese firm SZ DJI was one among the first commercial take offs in 2016 and further growth taken place with more commercial applications in insurance, construction and agriculture. The software tool SOJUMP was used to manage the data gathering process. The survey questionnaire was divided into two parts. In the first part, questions referred to people’s basic information, including gender, age, education level, working experience, company’s affiliation and other relevant information. The purpose for this part of the questionnaire was to develop some knowledge about the participants and their companies. In the second part of the survey, the questions were designed according to the Technology Acceptance Model. The purpose of these questions was to test the perception of drone-enabled delivery systems. In this section, all the answers used the 7-point LIKERT scale ranging from 1 (strongly disagree) to 7 (strongly agree), while number 4 represents a neutral attitude.

The research measures each construct through the following questions derived from the literature evaluation.

**Table 1:** Survey questions

Construct	Items	Variables
Efficient operations (adapted from Thompson et al., 1991)	EF1	Using drones to deliver packages may be more costly than other delivery methods.
	EF2	Delivery drones will not cost more for daily operations than other methods.
	EF3	With improved efficiency, more people will prefer drones.
Security (adapted from Vijayasarathy, 2004)	S1	Packages will be safe when using delivery drones for logistics.
	S2	In general, it can be trusted that personal privacy will be safeguarded through new delivery methods.
	S3	Delivery drones may have a strict security monitoring system.
Perceived usefulness (adapted from Davies, 1989)	PU1	Using drones will simplify the delivery process.
	PU2	Delivery drones will be more useful if the packages are less than 20 kg.
	PU3	Using delivery drones will substantially reduce the labour force.
Perceived ease of use (adapted from Davies, 1989)	PEOU1	My interaction with drones will be simple and straightforward.
	PEOU2	It will be easy to program drones to do what I want them to do.
	PEOU3	Training employees to use drones is simple.
Attitude (adapted from Thompson et al., 1991)	ATDU1	I would always look for opportunities to use delivery drones in my work place.
	ATDU2	I look forward to those aspects of my job that require me to use delivery drones.
	ATDU3	It is not essential for me to work with delivery drones.
Behavioural intention (adapted from Vijayasarathy, 2004)	BI1	I intend to use delivery drones to do my delivery work in the future if possible.
	BI2	I intend to use the delivery drones whenever appropriate to do my delivery work.
	BI3	If possible, I strongly believe this new technology will benefit the beneficiaries.

### Data collection

The commercial operations scaled up during 2016 where there were many queries related to operational factors, supply chain factors, organisational and behavioural factors. The findings from 2016 data would be useful key lessons for firms in the emerging countries who are at early stage to implement emerging innovations and successfully integrating those technologies without any impediments. Between May and August 2016, a total of 103 copies of questionnaire were completed by different logistics companies’ staff from 10 major Chinese logistics companies. Since the scope of the study is to understand the readiness of the operational processes in the early adopters where there were only a few logistics firms in China came forward to implement the drone technologies. Questionnaires were administered through the SOJUMP website. After checking, all data were assessed as valid and the samples used in the analysis. Basic participant information is shown in Table 2.

**Table 2:** Basic information of participants

Characteristics		Frequency	Percentage	Valid Percentage	Cumulative Percentage
**Gender**	Male	75	72.8	72.8	72.8
	Female	28	27.2	27.2	100.0
**Age**	18–29	52	50.5	50.5	50.5
	30–39	34	33.0	33.0	83.5
	40–49	14	13.6	13.6	97.1
	Above 50	3	2.9	2.9	100.0
**Education Level**	High school	35	34.0	34.0	34.0
	Bachelor’s degree	54	52.4	52.4	86.4
	Master’s degree	14	13.6	13.6	100.0
**Company**	YTO	10	9.7	9.7	9.7
	STO	11	10.7	10.7	20.4
	YUNDA	10	9.7	9.7	30.1
	ZTO	13	12.6	12.6	42.7
	S.F	10	9.7	9.7	52.4
	EMS	10	9.7	9.7	62.1
	TTK	9	8.7	8.7	70.9
	BEST	10	9.7	9.7	80.6
	JD	11	10.7	10.7	91.3
	DHL	9	8.7	8.7	100.0
**Experience in logistics**	1–3	48	46.6	46.6	46.6
	3–5	31	30.1	30.1	76.7
	5–10	15	14.6	14.6	91.3
	Above 10	9	8.7	8.7	100.0
**Position**	General staff	58	56.3	56.3	56.3
	Low-level manager	29	28.2	28.2	84.5
	Mid-senior manager	15	14.6	14.6	99.0
	Others	1	1.0	1.0	100.0
**Turnover per year (million Yuan)**	Less than 0.1	0	0	0	0
	0.1-1	0	0	0	0
	1–10	0	0	0	0
	Above 10	103	100	100	100
**Awareness of delivery drones**	Yes	91	88.3	88.3	88.3
	No	12	11.7	11.7	100.0
**Experience new technology**	Yes	38	36.9	36.9	36.9
	No	65	63.1	63.1	100.0
Total		103	100.0	100.0	

### Data analysis

We assessed for non-response bias by comparing early respondents to late respondents; the responses were split into two groups based on the when they were returned. We did not find any significant differences (95% confidence level) in the firm characteristics such as turnover, age and experience of employees. Additionally, we also compared the non-responding logistics firms to those that did not respond. Specifically, we randomly selected 10 logistics firms from the list of firms that did not participate in our survey and compared them to the respondent sample of 103. We did not find any significant differences (95% confidence level) in turnover, experience of employees and age between these groups. These results suggest that non-response bias need not be a major concern in our research.

According to Mansson (2015), reliability analysis is used to test the stability degree and the consistency of the results. Cronbach’s Alpha for each construct is more than 0.7, which shows the credibility of scale is acceptable. The values of constructs and items are shown in Table 3.

**Table 3:** Assessment of the model

Constructs	Variables	Standardized Factor Loadings	Cronbach’s alpha	Composite reliability	AVE
Efficiency	EF1	0.858	0.710	0.77	0.59
	EF2	0.999			
	EF3	0.214			
Security	S1	0.584	0.710	0.71	0.46
	S2	0.644			
	S3	0.788			
Perceived usefulness	PU1	0.501	0.780	0.79	0.57
	PU2	0.852			
	PU3	0.859			
Perceived ease of use	PEOU1	0.781	0.828	0.80	0.57
	PEOU2	0.843			
	PEOU3	0.639			
Attitude	ATDU1	0.832	0.825	0.78	0.56
	ATDU2	0.930			
	ATDU3	0.370			
Behavioural intention	BI1	0.767	0.858	0.83	0.62
	BI2	0.812			
	BI3	0.785			

The purpose of the survey instrument is to make the measurement and results reach a higher level of validity. A higher validity can reveal a higher fidelity of the measured behaviour (Tatham et al., 2017). Before the analysis of the exploratory factors, we tested whether the sample is suitable for this analysis, one of which is the validity analysis that can examine the correlation between the variables. In order to test the validity in this paper, it was necessary to put all the sub-scales into a Kaiser-Meyer-Oklin (KMO) test. According to the results of the test, it was found that the KMO value is 0.838, which means the result is accepted. In other words, it has a normal distribution and suitable for factor analysis (Topaloglu et al., 2016). In addition, the significance values were also checked.

The main criterion for assessing the final result is the value of factor loadings; these can be interpreted as a tool for examining the relationships among factors and variables (Gorsuch, 1983). In the study, all the variables were considered; finally the variables with factor loading values below 0.5 were deleted. Based on this we arrived at six factors with a minimum of three variables. Table 3 below shows the specific information related to reliability and validity analysis, including variables included in the analysis, factor loading for each variable, Cronbach’s alpha, composite reliability and average variance extracted (AVE), respectively. AVE is used to assess the discriminant validity, which measures the average variance shared between a construct and its measures (Thompson et al., 1991). If the value of AVE is over 0.5, it indicates good convergent validity. From the table, although the value of security is less than 0.5, it is extremely close to the requirement; this can be treated as acceptable. In terms of standardised factor loading, the couple of items (EF3 and ATDU3) loading are lower and still it is considered because of theoretical underpinning of the items acceptance from the beneficiaries perspective and specifically ATDU3 is a reverse worded item introduced to reduce acquiescence bias of respondents. Overall, all the factors in this paper pass the reliability and validity test and they can therefore be used for further analysis.

Previous studies have used structural equation modelling (SEM) to understand the direct and indirect effect of casual relationship between the latent variables such as adoption factors and behavioural intentions in different industry contexts such as manufacturing, logistics and technology commercialisation success which cant be directly measured (Yu et al., 2015; Nordhoff et al., 2021). Considering that structural equation modelling (SEM) allows the modelling of a set of relationships among constructs and a simultaneous estimation of all hypothesized paths, we decided to use SEM to test the hypotheses. In this study we followed a two-step approach ie.e constructing the measurement model first and then testing the structural model (Anderson and Gerbing 1988). AMOS (Fishbein, 1976) was used to conduct the SEM analysis. The guidance from Gefen et al. (2011) was used in this process. SEM is a simultaneous equation represented in matrices as shown $y=\beta y+\gamma x+ \epsilon$.

$${\rm{Where}}\,\,\left[ {\begin{array}{*{20}{c}}{y1}\\{}\\{yn}\end{array}} \right] = \begin{array}{*{20}{c}}0\\{\beta 21}\\{bn1}\end{array}\,\,\,\begin{array}{*{20}{c}}{\beta 21}\\0\\{bn2}\end{array}\,\,\,\begin{array}{*{20}{c}}{\beta 1n}\\{}\\0\end{array}\,\,\left[ {\begin{array}{*{20}{c}}{y1}\\{}\\{yn}\end{array}} \right]\,\,\, + \begin{array}{*{20}{c}}{\gamma 11}\\{\gamma 21}\\{\gamma n1}\end{array}\,\,\,\begin{array}{*{20}{c}}{\gamma 12}\\{\gamma 22}\\{\gamma n2}\end{array}\,\,\,\begin{array}{*{20}{c}}{\gamma 1p}\\{\gamma 2p}\\{\gamma np}\end{array}\,\,\,\,\left[ {\begin{array}{*{20}{c}}{x1}\\{}\\{xp}\end{array}} \right]\, + \,\,\,\left[ {\begin{array}{*{20}{c}}{\varepsilon 1}\\{}\\{\varepsilon n}\end{array}} \right]$$

Table 4 shows the correlation among all the items. Table 5 shows the model scores related to the goodness of fit; it also shows the standard value for examining. It is obvious that the chi-square value, which is 1.602, is less than the prescribed values. Then the next four indices, including comparative fit index (CFI), Tucker-Lewis (TLI), normed fit index (NFI) and incremental fit index (IFI), are all used to assess model fit. Overall the model fit indices in the paper meets the requirements. In addition, the root mean squared error of approximation (RMSEA) is less than 0.08, signifying that the outcome in the analysis achieves this goal.

**Table 4:** Correlation table

	EF3	ATDU3	EF2	EF1	S3	S2	S1	BI3	BI2	BI1	ATDU2	ATDU1	PEOU3	PEOU2	PEOU1	PU3	PU2	PU1
EF3	1.00																	
ATDU3	− 0.00	1.00																
EF2	0.21	− 0.01	1.00															
EF1	0.18	− 0.01	0.86	1.00														
S3	0.00	0.14	0.00	0.00	1.00													
S2	0.00	0.11	0.00	0.00	0.51	1.0												
S1	0.000	0.10	0.00	0.00	0.46	0.38	1.00											
BI3	− 0.002	0.25	− 0.01	− 0.01	0.24	0.20	0.18	1.0										
BI2	− 0.00	0.26	− 0.01	− 0.01	0.25	0.20	0.18	0.64	1.00									
BI1	− 0.00	0.25	− 0.01	− 0.01	0.24	0.19	0.17	0.60	0.62	1.00								
ATDU2	− 0.00	0.34	− 0.02	− 0.02	0.34	0.28	0.25	0.63	0.65	0.62	1.0							
ATDU1	− 0.00	0.31	− 0.02	− 0.02	0.30	0.25	0.23	0.57	0.58	0.55	0.77	1.00						
PEOU3	0.00	0.20	0.00	0.00	0.26	0.21	0.19	0.36	0.37	0.35	0.5	0.44	1.00					
PEOU2	0.00	0.26	0.00	0.00	0.34	0.28	0.25	0.48	0.49	0.47	0.66	0.59	0.54	1.00				
PEOU1	0.00	0.24	0.00	0.00	0.32	0.26	0.23	0.44	0.46	0.43	0.61	0.54	0.5	0.66	1.00			
PU3	− 0.01	0.22	− 0.07	− 0.06	0.29	0.24	0.22	0.36	0.37	0.35	0.55	0.49	0.34	0.44	0.41	1.00		
PU2	− 0.01	0.22	− 0.06	− 0.06	0.29	0.24	0.22	0.36	0.37	0.35	0.54	0.48	0.33	0.44	0.41	0.73	1.00	
PU1	− 0.01	0.13	− 0.04	− 0.03	0.17	0.14	0.13	0.21	0.22	0.21	0.32	0.29	0.2	0.26	0.24	0.43	0.43	1.00

**Table 5:** Fit indices of SEM Analysis

Model (N = 103)	Df	χ2	χ2 /df	NFI	IFI	TLI	CFI	RMSEA(%)
Model score	128	205.05	1.602	0.805	0.916	0.900	0.914	0.077

Note: SEM = structural equation model; NFI = normed fit index; IFI = incremental fit index; TLI = Tucker-Lewis index; CFI = comparative fit index; RMSEA = root mean square error approximation

### Findings

We tested the hypothesis using structural equation modelling (SEM). Path co-efficient for various relationships is shown in Table 6. The results show that the coefficient of the path between efficiency and perceived usefulness is not significant. Therefore, Hypothesis 1 is not supported. Likewise, the significance of the coefficient between security and perceived usefulness is not significant, thus rejecting Hypothesis 2. Hypothesis 3 makes an assumption that security will have a positive effect on the perceived ease of use. It can be concluded that this hypothesis is supported as the coefficient of the path is highly significant, as indicated by the ‘p’ value. The result also indicates that the perceived ease of use will have a positive influence on perceived usefulness due to the low coefficient. Therefore, Hypothesis 4 is accepted. Similarly, Hypotheses 5, 6 and 8 are accepted. Finally, the coefficient of the path between perceived usefulness and behavioral intention to use is not significant; therefore, Hypothesis 7 is rejected.

**Table 6:** Results of the hypotheses using SEM

Relationship		S.E	Path Coefficients	T	P-value	Results
H1: Perceived usefulness ← Efficiency		0.04	-0.08	− 0.82	0.410	Unsupported (*The efficiency of using delivery drones will significantly influence the perceived usefulness of the new technology*)
H2: Perceived usefulness ← Security		0.21	0.16	1.17	0.241	Unsupported (*The security of using delivery drones will significantly influence the perceived usefulness of the new technology*)
H3: Perceived ease of use ← Security		0.24	0.51	3.53	0.000	Supported (*The security of using delivery drones will significantly influence the perceived ease of use of the new technology*)
H4: Perceived usefulness ← Perceived ease of use		0.16	0.53	3.22	0.001	Supported (*The perceived ease of use of delivery drones will significantly influence the perceived usefulness of the new technology*)
H5: Attitude towards use ← Perceived usefulness		0.17	0.28	2.38	0.017	Supported (*The perceived usefulness of delivery drones will significantly influence attitudes towards new technology use*)
H6: Attitude towards use ← Perceived ease of use		0.17	0.67	5.39	0.000	Supported (*The perceived ease of use of delivery drones will significantly influence attitudes towards new technology use*)
H7: Behavioural intention to use ← Perceived usefulness		0.13	-0.10	− 0.82	0.410	Unsupported (*The perceived usefulness of delivery drones will significantly influence the behavioural intention of new technology use*)
H8: Behavioural intention to use ← Attitude towards use		0.11	0.94	6.18	0.000	Supported (*Attitude towards delivery drone use will significantly influence the behavioural intention of new technology use*)

*** Significant at the 0.001 level (two-tailed).

## Discussion

Our model hypothesizes that from the perspective of Chinese logistics companies, efficiency and security can be key drivers of the intention to use new delivery systems. However, our empirical analysis suggests that ease of use and security are most significant in shaping attitudes to the adoption of drone delivery for humanitarian operations. This suggests that, currently, drone delivery systems are not seen as potentially cost reducing innovations that can deal with conventional bulk delivery challenges; however, they can be used for specialist delivery scenarios such as humanitarian operations, if the security issues are addressed. In terms of operational process the respondents didn’t realise the benefits this emerging technology can bring in because of these technology adoption don’t follow linear patterns where there needs to be ongoing experimental iterative process in the use of technology before they get much clarity in terms of efficiency (Maghazei et al., 2022). The problem of establishing the beneficiary or recipient’s identity remains a key issue. It may be that there is also some intention to use drone delivery based on the initial novelty value of the technology, but unless ease of use is achieved and security issues are sorted, drone delivery would not develop as a permanent and standard service. A key question here is under what circumstances could drone-enabled delivery systems create efficiency and effectiveness of humanitarian logistics operations. We propose that the ability to deliver emergency needed packages quicker than conventional delivery systems is the value proposition here. This value proposition increases as the delivery eco-system becomes more challenging for conventional transport, for example, difficult to access rural or heavily congested urban locations, which are affected by emergency situations.

Our findings are consistent with some of the most recent developments in drone delivery systems in which drones are used to deliver specialist medical packages in very difficult landscapes. Innovative companies, such as Zipline (founded in 2014), have tested the delivery of medical supplies such as blood and medicines to hospitals in Rwanda. Similarly, other start-ups have sought to develop similar systems; these include Matternet, which is working to deliver medical testing kits with drones, and Flirtey, which delivered drugs to a medical centre in rural Virginia last year — the first FAA-approved delivery of its kind. Furthermore, one of the major Chinese logistics companies involved in our study, SF, carried out its first demonstration of using drones to deliver emergency supplies in December 2017. This was an attempt to promote the development of the use of drone in the whole logistics industry. It is interesting to note that some logistics companies view humanitarian operations as a context to experiment and test drone delivery systems that they could then use in their commercial operations. It would be important to explore this issue further in the future.

### Theoretical implications

This study explains the sufficiency of fundamental acceptance theories in understanding the nuances of the use of emerging technologies with respect to the context of uncertainty in the occurrence of events and use of technology in a particular sector. Typically, the extraction of constructs from the three basic versions of acceptance models (such as Theory of Reasoned Action, Theory of Planned Behaviour and Technology Acceptance Model), with the current digital era’s challenges, including efficiency and safety, will benefit the scholarly community in testing the future emerging technological applications. The integration of constructs from basic acceptance theories and the evolving digital technological constructs are the major theoretical contributions of this study. Specifically the study offers empirical evidence to understand the operational factors, supply chain factors and behavioural factors with respect to drone technology usage in the context of humanitarian operations. Even though there are confusing insights from the previous survey based studies on the adoption of emerging technologies this study in particular reveals some useful thoughts for future researchers to move forward in developing typical research characteristics through experimentation to understand the role of efficiency, security and behavioural intention of employees who are engaging with the drone technology.

### Managerial implications

The empirical study focuses on logistics service providers in China and the intention of their staff to adopt delivery drones for humanitarian operations; our findings and insights developed here are relevant beyond this context. Also the study addresses the research direction mentioned by Wamba (2020) on the use of robot and drone to disaster relief operations from the engagement of 3rd party logistics service providers. The paper is useful to logistics managers and others who are considering the use of delivery drones, presenting the opportunities and synergies that the introduction and further understanding of these systems can create across commercial and humanitarian sectors.

Overall, we provided an overview of the development trajectories for the adoption of drone by logistics services providers, and a systematic analysis of the opportunities and remaining challenges involved. The paper explains what role drone systems can play in humanitarian operations and, more generally, how the use of delivery drones can improve the agility of both commercial and humanitarian operations, especially in densely populated urban areas and remote rural environments. It also highlights current limitations that need to be taken into account and overcome.

## Conclusions

Our review of previous studies of drone delivery systems in commercial and humanitarian contexts highlights their technological and institutional limitations (such as vulnerability to hacking and theft, inability to identify recipients, lack of durability and flying limitations, interference with commercial aircrafts and regulation), as well as the potential in the specialist delivery of high-frequency, low-weight items. Although significant barriers to developing the new technology still remain, drone offer new opportunities for 3PLs to improve the effectiveness and agility of humanitarian operations.

This paper explored the relative importance of user perception of the key factors affecting the adoption of drones as a delivery system by 10 major Chinese logistics companies. A research model based on technology acceptance theories and models was developed and applied. We found that behavioural intention to use drones is determined by personal attitudes, and that these attitudes are predominantly shaped by the perceived ease of use rather than perceived efficiency. Security is a key factor that influences the perception of ease of use. Participants are concerned more about whether a serious monitoring system can be developed, which is not only related to the safety of the drone and package, but also refers to other privacy issues. Whether autonomous delivery drones can be easily controlled is another key factor under the perceived ease of use factor. Using drones in humanitarian contexts will require the expertise and capacity to analyse large volumes of aerial image data. Air traffic management and data privacy issues also create regulatory and ethical challenges (Luterbacher, 2018). Nevertheless, the results show that the logistics companies should be encouraged to consider the use of drones for humanitarian operations as a strategic initiative. They should conduct some pilot tests involving the logistics planners and the recipient of goods to test and validate the ease of usage and allay any security concerns.

There are some limitations in this study. First, it has proven challenging to involve a wide range of senior managers in the logistics companies. Second, the study would have benefited from more participants than the 103 from 10 major Chinese logistics companies involved. Future research should explore the issues involved in the use of drone delivery through a focus on decision-makers across different commercial and humanitarian logistics application areas (i.e., e-commerce deliveries), and different types of humanitarian logistics operations (i.e., mapping and monitoring, delivery of supplies, and search and rescue efforts for both natural and man-made disasters while distinguishing between urban and rural areas). Such detailed analysis can generate insights into the most appropriate application areas of use of drones in humanitarian operations considering the performance requirements and the incurred costs, to identify barriers in specific application areas and overcoming those barriers. Finally, as the application of drones is rapidly evolving, researchers should engage in longitudinal studies of technology acceptance to capture the changes over a period of time.
